# Two-stage mass spectrometry approach for the analysis of triterpenoid glycosides in *Fagonia indica*†

**DOI:** 10.1039/c8ra08350a

**Published:** 2018-12-07

**Authors:** Nayab Kanwal, Amna Jabbar Siddiqui, Faraz Ul Haq, Hesham R. El-Seedi, Syed Ghulam Musharraf

**Affiliations:** H.E.J. Research Institute of Chemistry, International Center for Chemical and Biological Sciences, University of Karachi Karachi-75270 Pakistan musharraf1977@yahoo.com + 92 213 4819018-9 +92 213 4824924-5 +92 213 4819010; Department of Medicinal Chemistry, Uppsala University, Biomedical Centre Box 574 SE-75 123 Uppsala Sweden

## Abstract

Triterpenoid glycosides are molecules widely distributed in plants and have shown a wide range of biological activities against various diseases. This paper describes the qualitative and quantitative analysis of triterpenoid glycoside (saponins) using a two-stage mass spectrometry approach in five samples of *Fagonia indica* collected from various parts of the country. In the first stage, triterpenoid glycosides were identified using liquid chromatography high-resolution mass spectrometry using UHPLC-QTOF-MS system. In the second stage, compounds were quantified using a multiple reaction monitoring (MRM) approach using an UHPLC-QQQ-MS system. *Fagonia indica* has shown a wide range of biological activities and found to be rich in saponin or triterpenoid glycoside constituents. A total of thirteen triterpenoid saponins were identified based on high-resolution analysis, MS/MS and database comparison, while six of them were simultaneously quantified using the multiple reaction monitoring (MRM) approach. The results indicate that the samples share a similar UHPLC pattern, however, the amount of these saponins in samples varies greatly. Compound 4*i.e.* nayabin D was the major constituent (1.4–3.8 μg g^−1^) among the six analyzed compounds. The results demonstrated that the developed multi-compound determination in combination with a fingerprint analysis method is rapid, accurate, precise and sensitive and can be utilized for quality control and high-throughput quantification of various saponins in *Fagonia indica* may be extended to other plant species.

## Abstract 2

Development of a rapid, accurate, precise, and effective method for the analysis and quantification of saponins in crude extracts from *Fagonia indica.*

## Graphical abstract

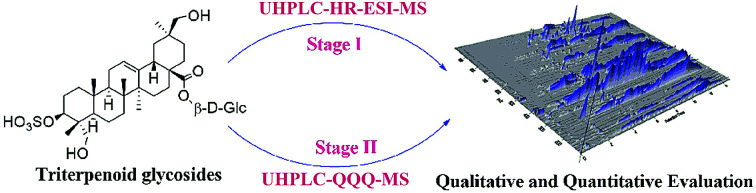

## Introduction

*Fagonia indica* belongs to family Zygophyllaceae and is scattered in subtropical, tropical, and temperate areas of the world including Algeria, Cyprus, Egypt, India, Morocco, Pakistan, and Saudi Arabia.^1^ Genus *Fagonia* with a variety of its species is also claimed to have medicinal properties which were investigated by researchers worldwide.^2,3^*Fagonia* species are often used in traditional medicines, for treating fever, jaundice,^4^ blood purification, cold, cough,^5^ asthma, skin infections, liver troubles,^6^ carminative and emetic conditions.^7^ Pharmacological effects of *Fagonia* species including antimicrobial,^8–10^ anti-inflammatory,^11^ antioxidant,^12^ antipyretic,^13^ analgesic,^14^ antitumor^8,15^ and anticancer^16^ properties have been well verified.

*Fagonia* species have obtained an enormous importance in phytochemical analysis over several years and have saponin or triterpenoid glycoside rich constituents. Saponin is one of a diverse class of secondary metabolites that are present commonly in the kingdom of plants.^17^ The complexity in the structure of saponins is imitated in the diversity of its pharmacological, biological, and physicochemical properties which leads to saponins as commercially important compounds with potential and an extensive diversity of usage in the food, cosmetics and pharmaceutical/healthcare industries. Some saponins from *Fagonia* species have been reported to exert anticancer and antioxidant activities.^18,19^ One of the compounds isolated from *Fagonia indica* exerts glucose-dependent insulin secretory activity, which seems to exhibit a decreased risk of drug-induced hypoglycemia and may offer distinct advantages as an anti-diabetic agent.^20^

UHPLC coupled with high-resolution mass analyzers like quadrupole-time-of-flight (QToF) combines the quick separation capability of liquid chromatography (LC) in conjunction with the higher resolving power of mass spectrometry (MS). However, use of a low-resolution mass analyzer like QQQ offers the quantitative analyses of analytes with high sensitivity. Combination of these two approaches ultimately increases the accuracy and specificity of the analytical results. This combination is widely used in qualification and quantification of glycosides in the different parts of many plants.^21–23^ The present study focuses on the chemical fingerprinting of saponins and simultaneously quantification using QToF and QQQ mass spectrometric approaches. The developed method was effectively applied to five samples of *Fagonia indica* collected from different locations for the first time. However, limited analysis of this herb has been reported so far.^24^

## Material and methods

### Reagents and materials

Six saponin standards, nayabin A–E (1–5), β-d-glucopyranosyl 3β-hydroxy-23-*O*-β-d-glucopyranosyloxy-taraxast-20-en-28-oate (6), were isolated from *F. indica* and characterized by spectroscopic studied.^20^ The purity of all standards was checked by HPLC and found to be >99% pure (peak area normalization). Glycyrrhizin used as internal standard was purchased from Sigma Aldrich (Darmstadt, Germany). Milli-Q water (Millipore, Bedford, USA) used in UHPLC analysis. Analytical grade formic acid and methanol were acquired from Fisher Scientific (Leicestershire, UK) and Merck (Darmstadt, Germany), respectively. All the sample solutions were filtered through a 0.45 μm PTFE membrane (Agilent Technology, China) prior to analysis. Plant materials of *Fagonia indica* were collected from different regions of Pakistan (S1–S5), mentioned in Table 4. Botanical authentication and identification of all samples was done from the Department of Botany, University of Karachi (UoK), Pakistan.

### Preparation of standard solutions

**Fig. 1:**
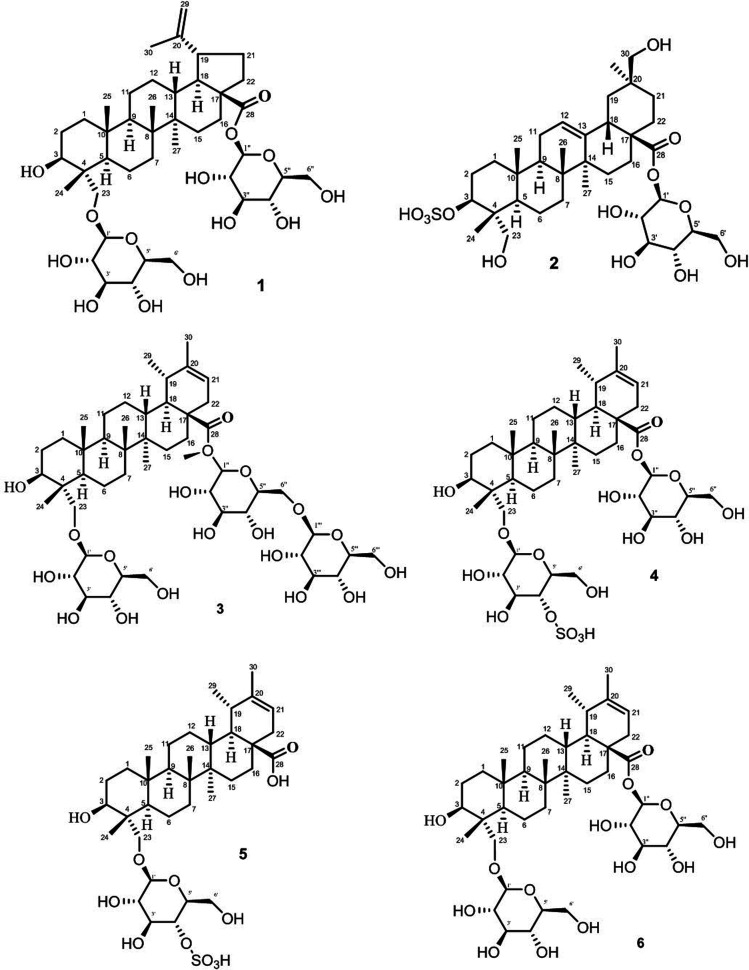
Structure of standard saponins 1–6 analyzed.

The stock solution was prepared by accurately weighing 0.5 mg of standards (1–6) and internal standard (I.S), dissolving them in 1 mL of 70% (v/v) methanol. Structures of all standards are shown in Fig. 1. The stock standard solutions were further diluted with 70% (v/v) methanol in order to provide six different concentrations for the establishment of calibration curves.

### Sample preparation

200 mg of air-dried (25–30 °C) various plant samples of *F. indica* were extracted with 70% methanol (20 mL) three times. Extraction was performed for 30 min on a shaker, subsequently 45 min on sonication bath. Each extract solution was combined and evaporated to dryness at 45 °C. At the time of analysis, 2 mL of 70% HPLC grade methanol was used for reconstitution of the dried extracts. The solutions were then filtered through 0.45 μm filters. The filtrates were diluted 10 folds at the time of analysis.

For spiking and recovery studies, known concentration of each standard was added to every sample prior to extraction. Three additional concentrations of standards were named as SP1, SP2 and SP3 which represented additional 200, 400 and 600 ng mL^−1^ of standards, respectively.

### Instrumentation and chromatographic conditions

Chromatographic separation was performed on Agilent Zorbax SB-18 MS column (3.0 × 50 mm i.d. 1.8 μm). The mobile phase was a binary gradient system prepared from 0.1% aqueous formic acid (eluent A) and 0.1% formic acid in methanol (eluent B), properly filtered and degassed for 15 min in an ultrasonic bath before to use. The mobile phase program was: 0–3 min, 10 to 65% B; 3–6 min, 65% B; 6–9 min, 65 to 90% B; 9–10 min, 90% B and 10–12 min, 90 to 10% B. The total run time was 13 min, including a 1 min equilibration time for the next run. The flow rate was at 0.5 mL min^−1^ and the injection volume was 5 μL. The column temperature was set at 35 °C.

An Agilent 1260 liquid chromatograph (Agilent Technologies, Wilmington, DE) equipped with an electrospray ionization (ESI) and operating under negative ion mode, on Qq-TOF-MS/MS instrument (QSTAR XL mass spectrometer Applied Biosystem/MDS Sciex, Germany) was used for the qualitative analysis of saponins. The data were recorded *via* information-dependent acquisition (IDA) experiments with the three most abundant ions in a mass spectrum. Precursor ion scans were recorded between 50 to 1100 *m*/*z* on a QSTAR XL mass spectrometer. The ESI interface conditions were as follows: curtain gas flow rate 20 L min^−1^, ion spray capillary voltage of 5500 V, focusing potential of 265 V, and nebulizer gas flow rate 30 L min^−1^, DP1 60 V, DP2 10 V. For MS/MS analysis, 20 to 45 eV collision energy was swept. Nitrogen gas delivered from Peak Scientific nitrogen generator was used as the curtain gas and collision gas. 3D images were formed through MZmine 2 open-source software. Compounds were identified with the help of high-resolution analysis, MS/MS analysis and database comparison.

The quantitative analysis was performed on an Agilent 1260 liquid chromatograph equipped with mass spectrometer possessed an ESI source and Agilent 6400 triple quadrupole (Agilent Technologies, Wilmington, DE, USA). MRM in negative mode was used for MS analysis. Several MS parameters were optimized as: drying gas is nitrogen at a flow rate of 8 L min^−1^ with a temperature of 320 °C, fragmentor voltage 125 V, a nebulizer pressure of 40 psi, and an electrospray capillary voltage of 3000 V. However, collision energy was attuned concerning every analyte separately in order to increase the analyte response.

## Results and discussion

### Saponin fingerprinting of *Fagonia indica* samples

Saponins of *F. indica* were analyzed by reversed-phase UHPLC-ESI-MS, with gradient mobile phase consisting of 0.1% formic acid in methanol and 0.1% aqueous formic acid. All analytes were eluted within 13 min including around 1 min equilibration time. Total ion chromatograms (TIC) of different located samples (S1–S5) of *Fagonia indica* are presented in ESI Fig. S1.† An overview of the TICs showed that the pattern of peaks eluted are similar, however their intensities may differ at some positions. 3D image analysis of TIC chromatogram of *Fagonia* sample was formed through software MZmine (ESI Fig. S2†), which represent the retention time on the *x*-axis, the mass-to-charge ratio (*m*/*z*) on the *y*-axis, and peak intensity on the *z*-axis. Maximum compounds are found in mass range of *m*/*z* 350–400, while most of the compounds are eluted in between the retention time (RT) of 2–11 min.

**Fig. 2:**
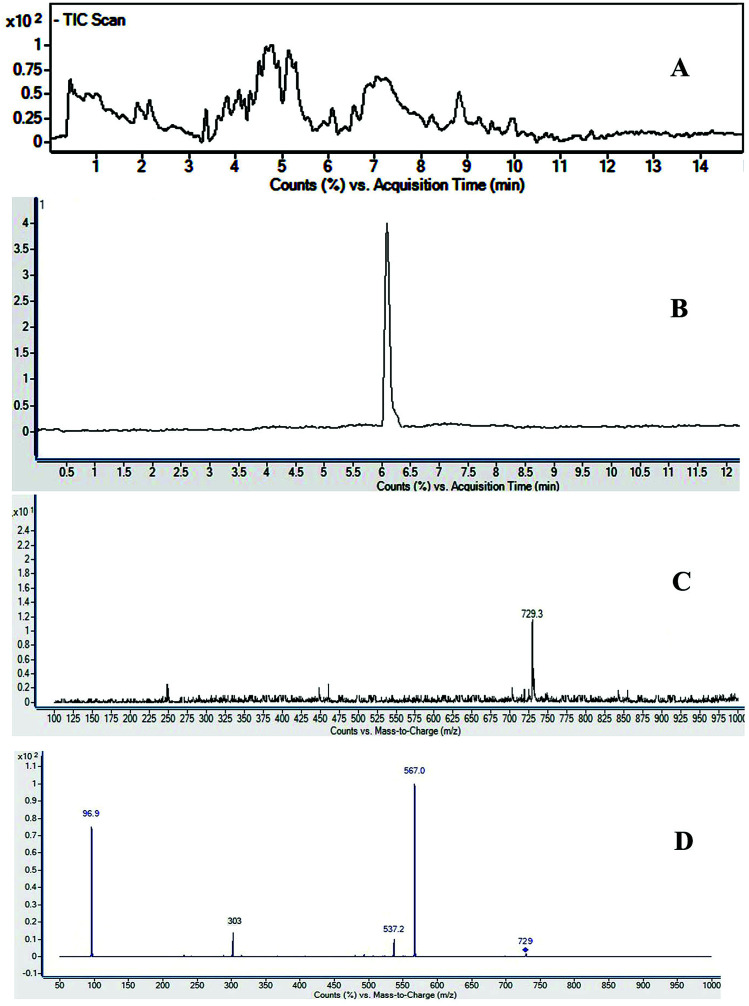
UHPLC-MS/MS spectra of *Fagonia indica* (A) total ion chromatogram (TIC), (B) extracted ion chromatogram (XIC) of *m*/*z* 729, (C) TOF-MS at retention time (RT) 5.6 min, and (D) MS/MS spectra of *m*/*z* 729.

**Scheme 1:**
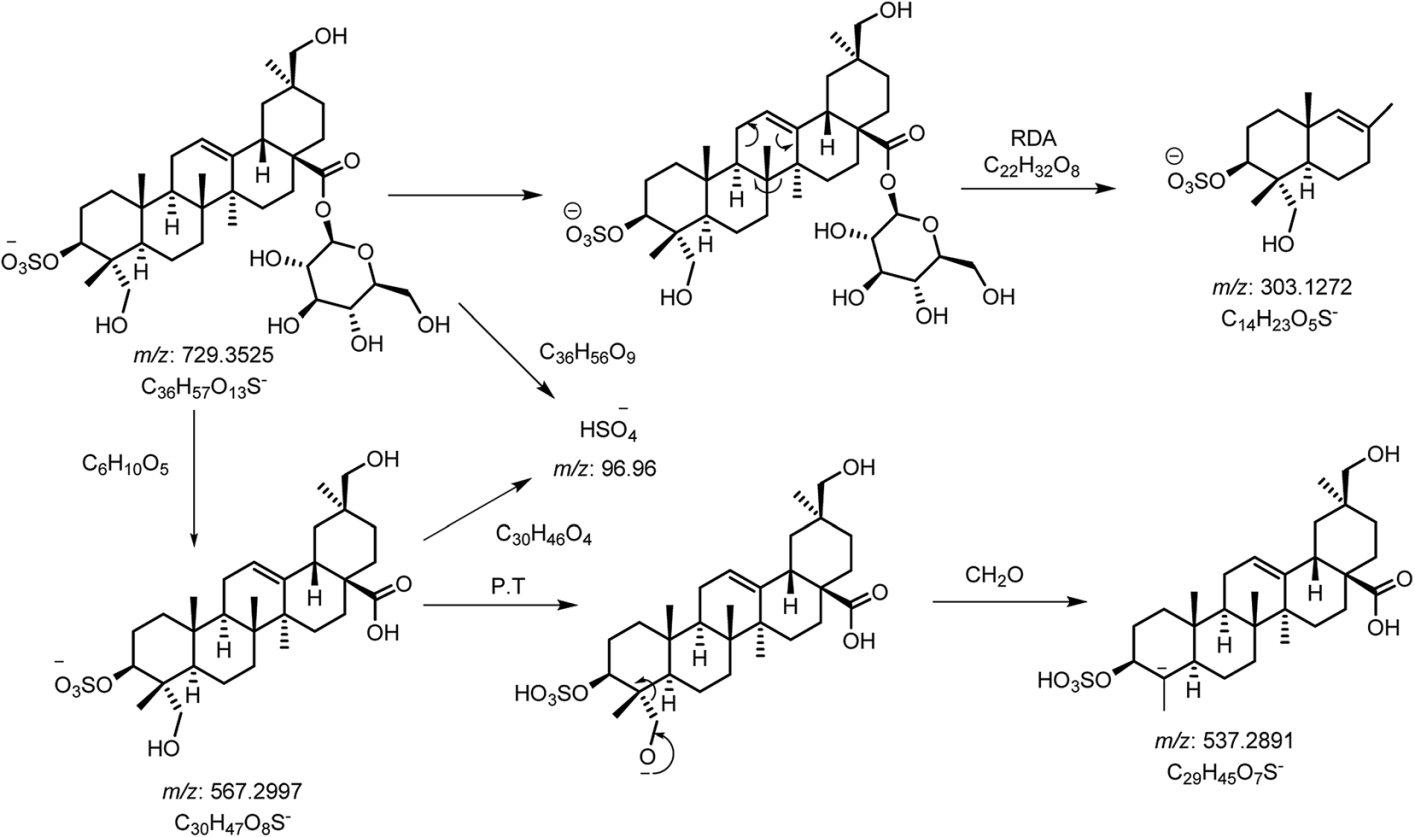
Proposed CID-MS/MS fragmentation pathway of compound 2 (*m*/*z* 729).

The full TIC scan in negative mode was used in the first step of identification. Each peak was first extracted from TIC as extracted ion chromatogram (EIC). At the second stage, MS/MS spectra were recorded for each compound using Independent Data Acquisition (IDA) experiment (Fig. 2). The MS/MS data of standard saponins (1–6) were recorded and their diagnostic ions and losses were identified, which were helpful for the identification of other saponins. ESI-QqTOF-MS/MS (negative mode) data of compound 2 is discussed below as a representative of saponin that showed [M − H]^−^ peak at *m*/*z* 729.3496 corresponding to the molecular formulae C_36_H_57_O_13_S (calc. 729.3519). Fragment ion at *m*/*z* 567 [M − H − 162]^−^ from *m*/*z* 729 due to the loss of one glucose moiety. Fragment at *m*/*z* 303 was supposed to be generated from *m*/*z* 729 by retro Diels–Alder cleavage of ring C, and fragment at *m*/*z* 537 was observed by the loss of CH_2_O from *m*/*z* 567. Another product ion at *m*/*z* 97 was indicating the fragment of sulfate group [HSO_4_]^−^. The mechanistic fragmentation pathway of compound 2 is presented in Scheme 1. ESI-QqTOF-MS/MS data of other standards were found to be similar. With the help of HR-ESI-MS analyses, isotopic distribution, MS/MS analysis of the [M − H]^−^ peaks, and database search using Dictionary of Natural Products (DNP), thirteen compounds including six isolated compounds were identified.^20,25–28^ Compound 1 showed its deprotonated molecule [M − H]^−^ at *m*/*z* 425.3825 corresponding to molecular formula C_30_H_50_O and was identified as taraxerol based on its accurate mass and MS/MS fragments. Compounds 3, 6, 7, 10, 11, 13 and 17 were identified based on their retention times, accurate masses, isotopic pattern and MS/MS fragmentation pattern matching with the standard compounds. Compound 8 appeared as [M − H]^−^ ion at *m*/*z* 713.3649 corresponding to molecular formula C_36_H_57_O_12_S and was identified as 3,27-dihydroxy-12-oleanen-28-oic acid; 3β-form, 3-sulfate, 28-*O*-β-d-glucopyranosyl ester based on its MS/MS fragmentation pattern. Compound 8 showed the loss of a glucose molecule at *m*/*z* 551. Compound 12 appeared as [M − H]^−^ ion at *m*/*z* 881.4955 corresponding to the molecular formula C_46_H_73_O_16_ and was identified as 3-*O*-β-d-xylopyranosyl (1→2)-[β-d-glucopyranosyl (1→3)]-α-l-arabinopyranosyl oleanolic acid based on MS/MS data. Compound 14 appeared as [M − H]^−^ ion at *m*/*z* 927.5025 corresponding to molecular formula C_47_H_75_O_18_ and showed a peak at *m*/*z* 765 corresponding to the loss of a glucose molecule while another peak appeared at *m*/*z* 517 which corresponds to the cleavage of rings B and C through retro-Diels–Alder reaction (RDA). Compound 14 was identified as 3-*O*-[β-d-glucopyranosyl (1→2)-α-l-arabinopyranosyl]-hederagenin, 28-*O*-β-d-glucopyranosyl ester. Compound 15 appeared as [M − H]^−^ ion at *m*/*z* 551.3055 corresponding to molecular formula 3,27-dihydroxy-12-oleanen-28-oic acid; 3β-form, 3-sulfate based on MS/MS data. Compound 16 showed its [M − H]^−^ ion at *m*/*z* 683.3521 corresponding to molecular formula C_35_H_55_O_11_S and identified as 3β-(2-*O*-sulfo-α-arabinopyranosyl)-27-dihydroxy, urs-12-en-28-oic acid based on its MS/MS data. The identities, retention times, molecular mass, molecular formula, and fragment ions for individual components are presented in Table 1.

**Table 1:** List of partially or fully identified compounds in the *Fagonia* extracts by UHPLC-MS and MS/MS

No	Name of compounds	RT (min)	Observed mass *m*/*z*	Ion type	Calc. mass [M − H]^−^	Mass accuracy (ppm)	Molecular formula	MS/MS (*m*/*z*)
1	Taraxerol	4.32	425.3825	[M − H]^−^	425.3861	−8.46	C_30_H_50_O	221
2	Unidentified	5.20	893.4225	[M − H]^−^	893.4205	2.24	C_42_H_69_O_18_S	731, 241, 139, 97
3	β-d-Glucopyranosyl 3β-*O*-sulfo-23,30-dihydroxy-olean-12-en-28-oate	5.60	729.3496	[M − H]^−^	729.3520	−3.29	C_36_H_57_O_13_S	567, 537, 303, 97
4	Unidentified	6.00	877.4299	[M − H]^−^	877.4256	4.90	C_42_H_69_O_17_S	715, 241, 97
5	3,27-Dihydroxy-12-ursen-28-oic acid; 3β-form, 3-*O*-(2-*O*-sulfo-α-l-arabinopyranoside), 28-*O*-β-d-glucopyranosyl ester	6.66	845.4008	[M − H]^−^	845.3993	1.77	C_41_H_65_O_16_S	683, 653, 637, 211, 139, 97
6	3β-Hydroxy-23-(2′,4′-di-*O*-sulfo-β-d-glucopyranosyloxy)-taraxast-20-en-28-oic acid	7.00	955.3645	[M − H]^−^	955.3667	−2.30	C_42_H_67_O_20_S_2_	875,713, 321, 241, 97
7	β-d-Glucopyranosyl-(1→6)-β-d-glucopyranosyl 3β-hydroxy-23-β-d-glucopyranosyloxy-taraxast-20-en-28-oate	7.07	957.4512	[M − H]^−^	957.5059	5.54	C_48_H_77_O_19_	633, 323, 221, 125
8	3,27-Dihydroxy-12-oleanen-28-oic acid; 3β-form, 3-sulfate, 28-*O*-β-d-glucopyranosyl ester	7.19	713.3649	[M − H]^−^	713.3571	10.93	C_36_H_57_O_12_S	683, 551, 97
9	Unidentified	7.70	875.4108	[M − H]^−^	875.4099	1.03	C_42_H_67_O_17_S	713, 477, 396, 255, 97
10	β-d-Glucopyranosyl 3β-hydroxy-23-β-d-glucopyranosyloxy-lup-20(29)-en-28-oate	8.11	841	[M + HCOOH − H]^−^	841.4586	−1.31	C_43_H_69_O_16_	633
11	β-d-Glucopyranosyl 3β-hydroxy-23-*O*-β-d-glucopyranosyloxy-taraxast-20-en-28-oate	8.73	841	[M + HCOOH − H]^−^	841.4586	−2.50	C_43_H_69_O_16_	633,323, 221, 125
12	3-*O*-β-d-Xylopyranosyl (1→2)-[β-d-glucopyranosyl (1→3)]-α-l-arabinopyranosyl oleanolic acid	8.80	881.4955	[M − H]^−^	881.4899	6.35	C_46_H_73_O_16_	835, 675
13	β-d-Glucopyranosyl 3β-hydroxy-23-(4′-*O*-sulfo-β-d-glucopyranosyloxy)-taraxast-20-en-28-oate	9.05	875.4066	[M − H]^−^	875.4099	−3.77	C_42_H_67_O_17_S	713, 471, 397, 241, 97
14	3-*O*-[β-d-Glucopyranosyl (1→2)-α-l-arabinopyranosyl]-hederagenin, 28-*O*-β-d-glucopyranosyl ester	9.28	927.5025	[M − H]^−^	927.4953	7.76	C_47_H_75_O_18_	765, 517,179
15	3,27-Dihydroxy-12-oleanen-28-oic acid; 3β-form, 3-sulfate	9.98	551.3055	[M − H]^−^	551.3043	2.18	C_30_H_47_O_7_S	521, 471, 97
16	3β-(2-*O*-Sulfo-α-arabinopyranosyl)-27-dihydroxy, urs-12-en-28-oic acid	9.42	683.3521	[M − H]^−^	683.3465	8.19	C_35_H_55_O_11_S	653, 637, 471, 97
17	3β-Hydroxy-23-(4′-*O*-sulfo-β-d-glucopyranosyloxy)-taraxast-20-en-28-oic acid	10.34	713.3570	[M − H]^−^	713.3571	−0.14	C_36_H_57_O_12_S	471, 397, 241, 97

### Optimization of mass spectrometric conditions for saponin quantification

**Fig. 3:**
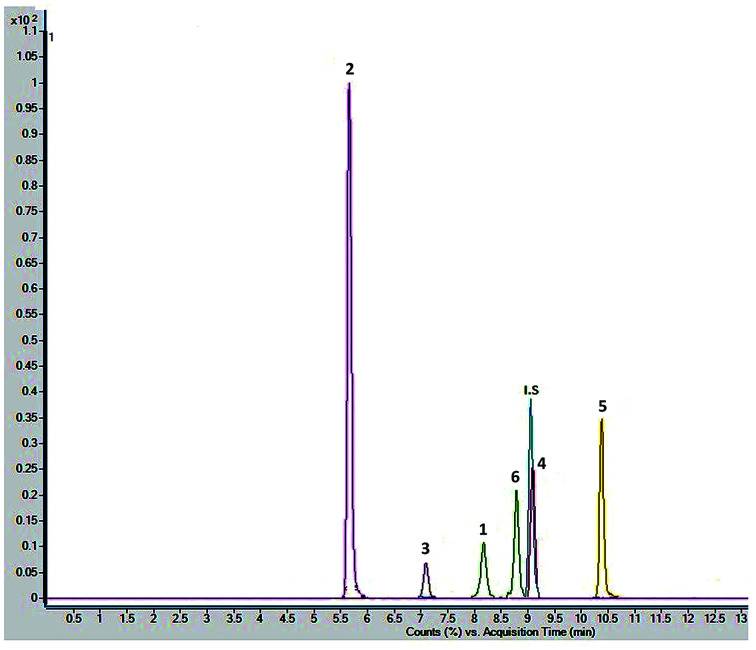
Combined chromatogram of standards 1–6 of *Fagonia indica* analyzed by MRM mode.

Chromatographic conditions of the mobile phase and gradient elution system were optimized in order to achieve good resolution and symmetric peak shapes of the six reference compounds in practical analysis time period. Chromatographic separation time was 13 min which also includes 1 min equilibration time. All compounds were eluted within 11 min. The MRM chromatographic spectra of six marker compounds and an internal standard are shown in Fig. 3. Both positive and negative modes of ionization were used for the investigation of all six marker compounds. However, the precursor and product ions were steady and reproducible in the negative mode. Hence, the negative mode of ionization was selected for further analysis.

**Fig. 4:**
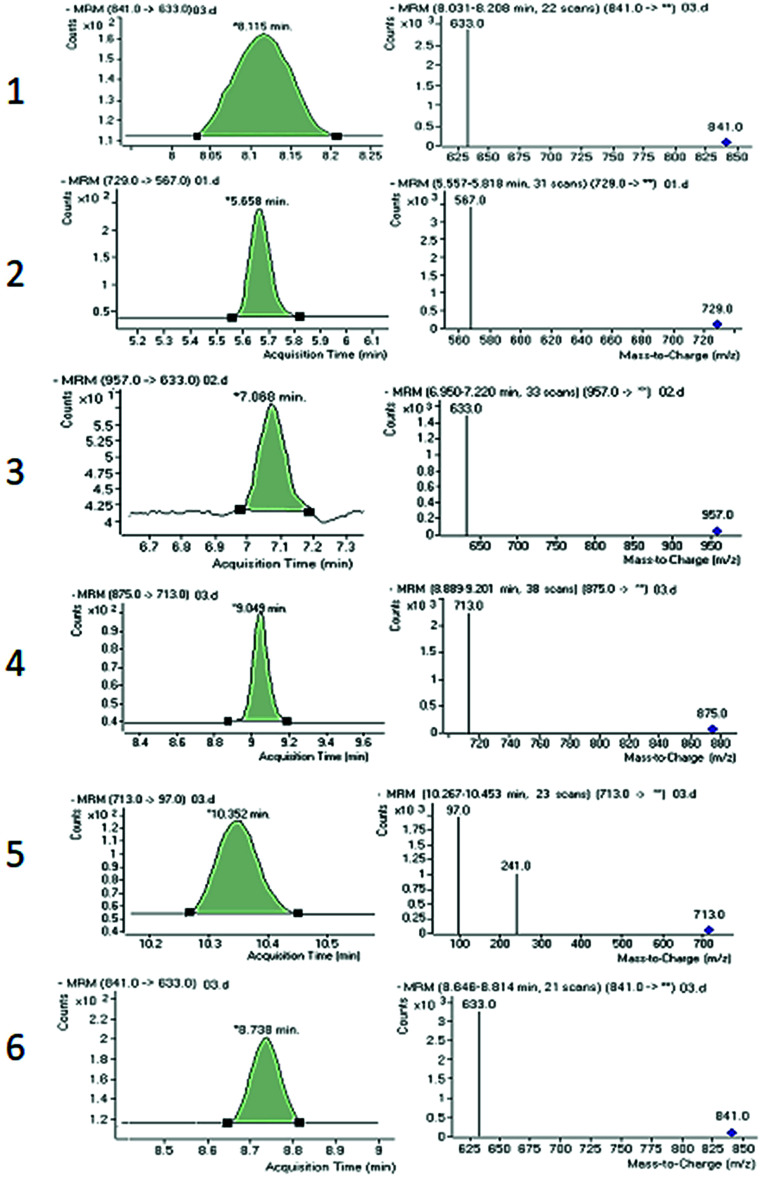
Summary of extracted ion chromatogram and product ion spectra of six analytes 1–6.

Both collision energy (CE) and fragmentor voltage (FV) play an important part in the process of fragmentation. Therefore, for obtaining more stable product ions and higher responses, collision energy and fragmentor voltage were optimized. Collision energy for the fragmentation of all analytes ranged from 40 to 80 V. Compounds 1, 2, 4, and 6 showed [M − H − 162]^−^ as product ion, as a result in the removal of one glucose molecule. While compound 5 showed [HSO_4_]^−^ and compound 3 showed [M − H − 162 − 162]^−^ due to the removal of two glucose moiety. These product ions were selected for quantitative MRM transition due to higher responses. A comprehensive list of all analytes including their retention times, optimized MS conditions and possible lost ions from the analytes is presented in Table 2. Summary of extracted ion chromatogram and product ion spectra of six analytes (1–6) are given in Fig. 4.

**Table 2:** Optimized MRM parameters for standards 1–6 of *Fagonia indica*

Analyte	Precursor ion mass (*m*/*z*)	Product ion mass (*m*/*z*)	Retention time (min)	Fragmentor voltage (V)	Collision energy (V)	Dwell time (ms)
1	841 [M − H + HCOOH]^−^	633 [M − H − 162]^−^	8.11 ± 0.02	125	40	60
2	729 [M − H]^−^	567 [M − H − 162]^−^	5.655 ± 0.006	125	70	60
3	957 [M − H]^−^	633 [M − H − 162 − 162]^−^	7.07 ± 0.01	125	60	60
4	875 [M − H]^−^	713 [M − H − 162]^−^	9.05 ± 0.01	125	80	60
5	713 [M − H]^−^	97 [HSO_4_]^−^	10.344 ± 0.007	125	80	60
6	841 [M − H + HCOOH]^−^	633 [M − H − 162]^−^	8.73 ± 0.01	125	40	60
IS	821 [M − H]^−^	351 [C_12_H_15_O_12_]^−^	9.04 ± 0.01	125	50	60

### Method validation

Various validation factors including the linear range (LR), reproducibility and repeatability (% RSD) at different levels of concentration, limit of detection (LOD), limit of quantification (LOQ), were assessed for the validation of the proposed procedure. Six concentration levels were analyzed three times for construction of the calibration curve. Every calibration curve was plotted based on the relative response of analyte to the internal standard (*y*) *versus* concentrations (*x*, ng mL^−1^). The calibration curves of compounds 1–6 are presented in the ESI Fig. S3.† The linearity response over the calculated range for all analytes was very good with varied correlation coefficients from 0.99818 to 0.99929 are shown in Table 3. The LOQs and LODs were calculated at approximate signal-to-noise ratio (S/N) of 10 and 3, respectively. The ranges for LOQ and LOD were obtained from 1.335 to 8.325 ng mL^−1^ and 0.440 to 2.747 ng mL^−1^, respectively. The results were also given in Table 3.

**Table 3:** Summary of calibration equations, limit of detection (LOD), and limit of quantitation (LOQ) data of the optimized method

Analyte	Linear calibration range (ng mL^−1^)	Regression equation	*R* ^2^	LOD (ng mL^−1^)	LOQ (ng mL^−1^)
1	250.00–1500.00	*y* = 5.09 × 10^−4^*x* − 2.05 × 10^−2^	0.99919	1.905	5.774
2	10.00–1500.00	*y* = 1.51 × 10^−3^*x* − 1.00 × 10^−2^	0.99929	0.387	1.175
3	250.00–1500.00	*y* = 1.12 × 10^−4^*x* + 4.42 × 10^−4^	0.99874	3.717	11.264
4	25.00–1500.00	*y* = 4.69 × 10^−4^*x* + 2.96 × 10^−3^	0.99904	2.039	6.180
5	250.00–1500.00	*y* = 5.77 × 10^−4^*x* − 1.03 × 10^−2^	0.99818	2.831	8.581
6	250.00–1500.00	*y* = 7.57 × 10^−4^*x* + 1.41 × 10^−1^	0.99912	4.336	13.140

The accuracy (%) and precision (% RSD) were evaluated by analyzing three different concentration levels in triplicate for all six saponins (ESI Fig. S4†). The precision calculation was divided into two parts: intra- and inter-day precisions. These were performed by repetitive injections on the similar day (intra-day) and on three consecutive days (inter-day) selected from the calibration range. In all the cases, accuracy was found to be in the range of 97.93 to 101.28% and precision was found below 3% RSD. The data of accuracy and precision of all standards are listed in ESI Table S1.†

The developed method was further used for simultaneous analysis of six marker compounds including non-sulfated triterpenoid and sulfated triterpenoid saponins in five *F. indica* samples from different regions of Pakistan (ESI Fig. S5†). Compound 4 was the major constituents among the five compounds analyzed, while other compounds also showed the high variations among five samples (Table 4). This high degree of discrepancy between the contents of five samples from different geographic locations could be due to several factors such as geographical origin, climate, time of harvest, and storage status.

**Table 4:** Analysis of six saponins in different samples of *Fagonia indica* (μg g^−1^)

Sample no.	Geographic distribution of sample	1	2	3	4	5	6
S-1	Hyderabad	0.219 ± 0.003	0.0081 ± 0.0002	0.067 ± 0.008	1.68 ± 0.04	0.844 ± 0.04	0.83 ± 0.04
S-2	New Sabzi Mandi, Karachi	0.253 ± 0.002	0.123 ± 0.001	0.0888 ± 0.0008	2.398 ± 0.007	0.563 ± 0.005	0.92 ± 0.04
S-3	Korangi, Karachi	0.425 ± 0.006	0.0027 ± 0.0002	0.16 ± 0.01	2.71 ± 0.05	1.271 ± 0.005	0.96 ± 0.01
S-4	University of Karachi, Karachi	0.223 ± 0.001	0.143 ± 0.002	0.0996 ± 0.0003	1.4427 ± 0.0002	0.3946 ± 0.0005	0.722 ± 0.001
S-5	Super Highway, Karachi	0.269 ± 0.007	0.16 ± 0.01	0.165 ± 0.001	3.8 ± 0.4	0.52 ± 0.03	2.47 ± 0.07

Results of recovery study at three additional concentrations (200, 400 and 600 ng mL^−1^) were found to be in the range of 90–110%. This shows that the developed method is accurate, precise and efficient for the analysis of saponins in complex plant matrix. Results of recovery study are presented in Table S2.†

## Conclusion

It has been demonstrated that the developed UHPLC-ESI-MS/MS method is rapid, accurate, precise, and effective for the analysis of saponins in crude extracts from *Fagonia indica*. Moreover, a procedure for the simultaneous quantification of six saponins in *Fagonia indica* has also been developed based on MRM approach. This approach is effective for quick identification of non-sulfated and sulfated triterpenoid saponins. Samples which were collected from five different locations, shared a similar chromatographic pattern; however, the concentration levels of the six characteristic saponins in the samples varied significantly. The newly developed UHPLC-ESI-MS/MS method can be useful for regular examination of saponins in botanicals and their formulations. Moreover, this two-stage mass spectrometry protocol can be applied for the efficient analysis of different classes of natural products in complex plant extracts.

## Conflicts of interest

The authors declare no competing financial interests.

## Supplementary Material

RA-008-C8RA08350A-s001
