# Supplementary material for: Two-stage mass spectrometry approach for the analysis of triterpenoid glycosides in Fagonia indica
Source: RSC Adv. 2018 Dec 7;8(71):41023–31. doi: 10.1039/c8ra08350a (PMC9091632; doi:10.1039/c8ra08350a)
Supplement: RA-008-C8RA08350A-s001 [file RA-008-C8RA08350A-s001.pdf]

Electronic Supplementary Material (ESI) for RSC Advances.
This journal is © The Royal Society of Chemistry 2018

Two-Stage Mass Spectrometry Approach for the Analysis of Triterpenoid Glycosides in *Fagonia indica*

Nayab Kanwal [a], Amna Jabbar Siddiqui [a], Faraz-Ul-Haq [a], Hesham R. El-Seedi [b], Syed Ghulam Musharraf [a,*]

[a] H.E.J. Research Institute of Chemistry, International Center for Chemical and Biological Sciences, University of Karachi, Karachi-75270, Pakistan.

[b] Department of Medicinal Chemistry, Uppsala University, Biomedical Centre, Box 574, SE-75 123, Uppsala, Sweden.

*Corresponding author. Tel.: +92 213 4824924-5; 4819010; fax: + 92 213 4819018-9.

E-mail address: musharraf1977@yahoo.com

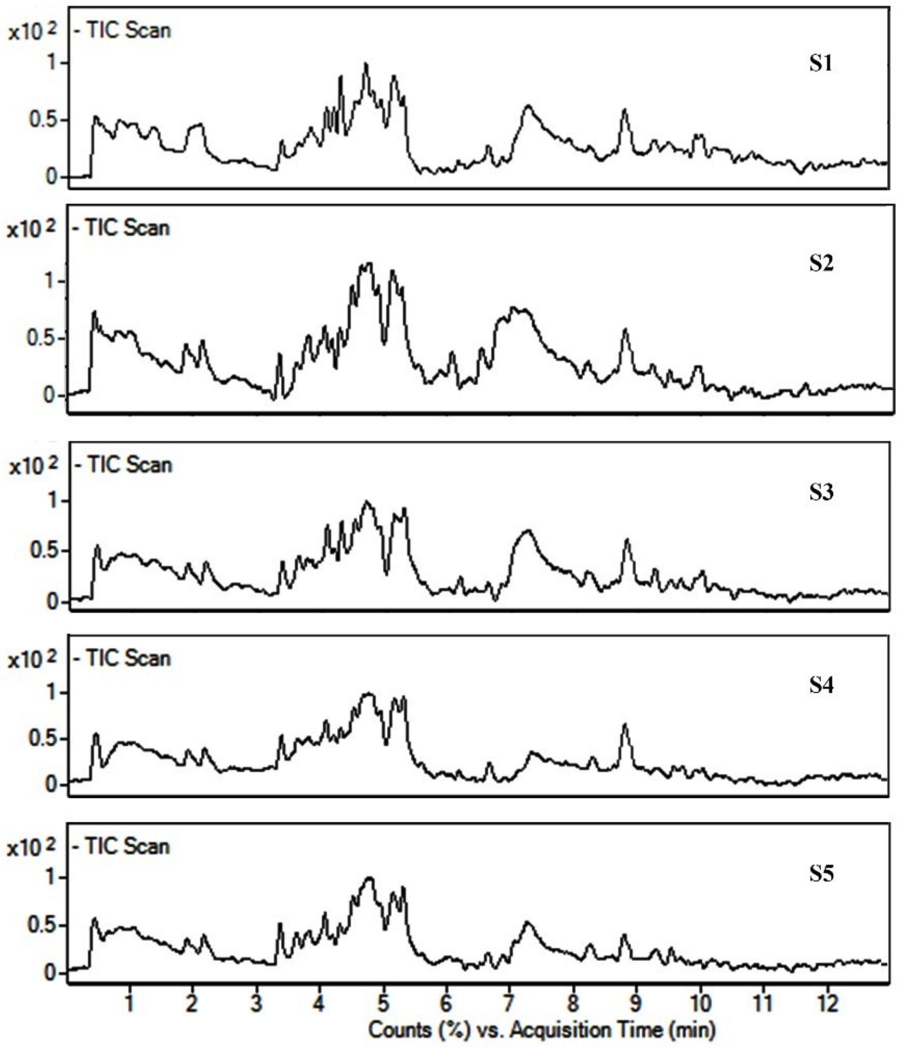

Figure S1: Comparison of TIC chromatograms of *F. indica* (S1-S5).

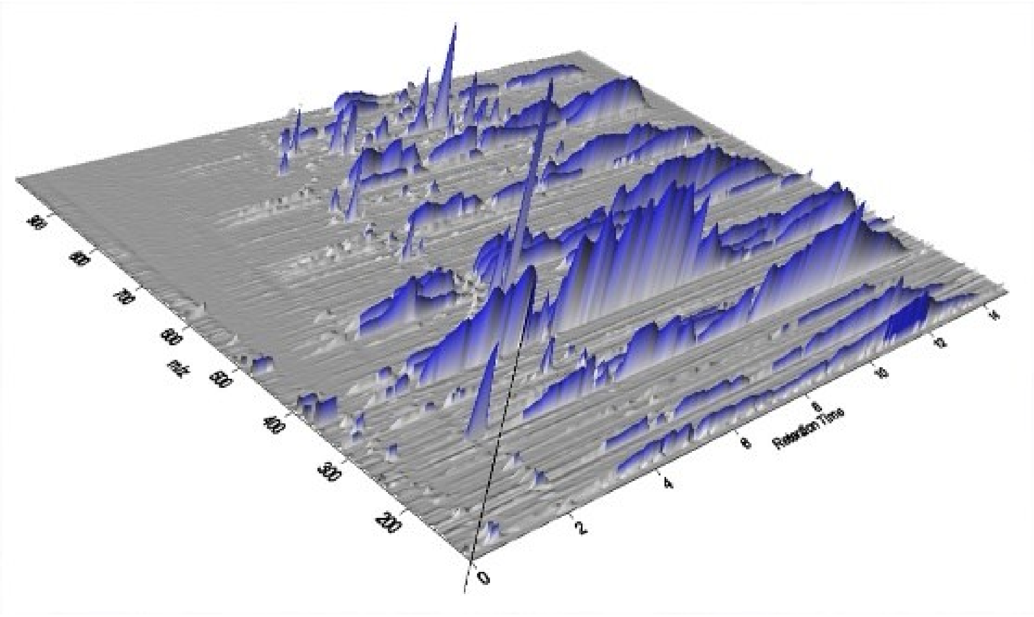

Figure S2: 3D Ion maps obtained in negative ionization mode of *Fagonia indica* sample.

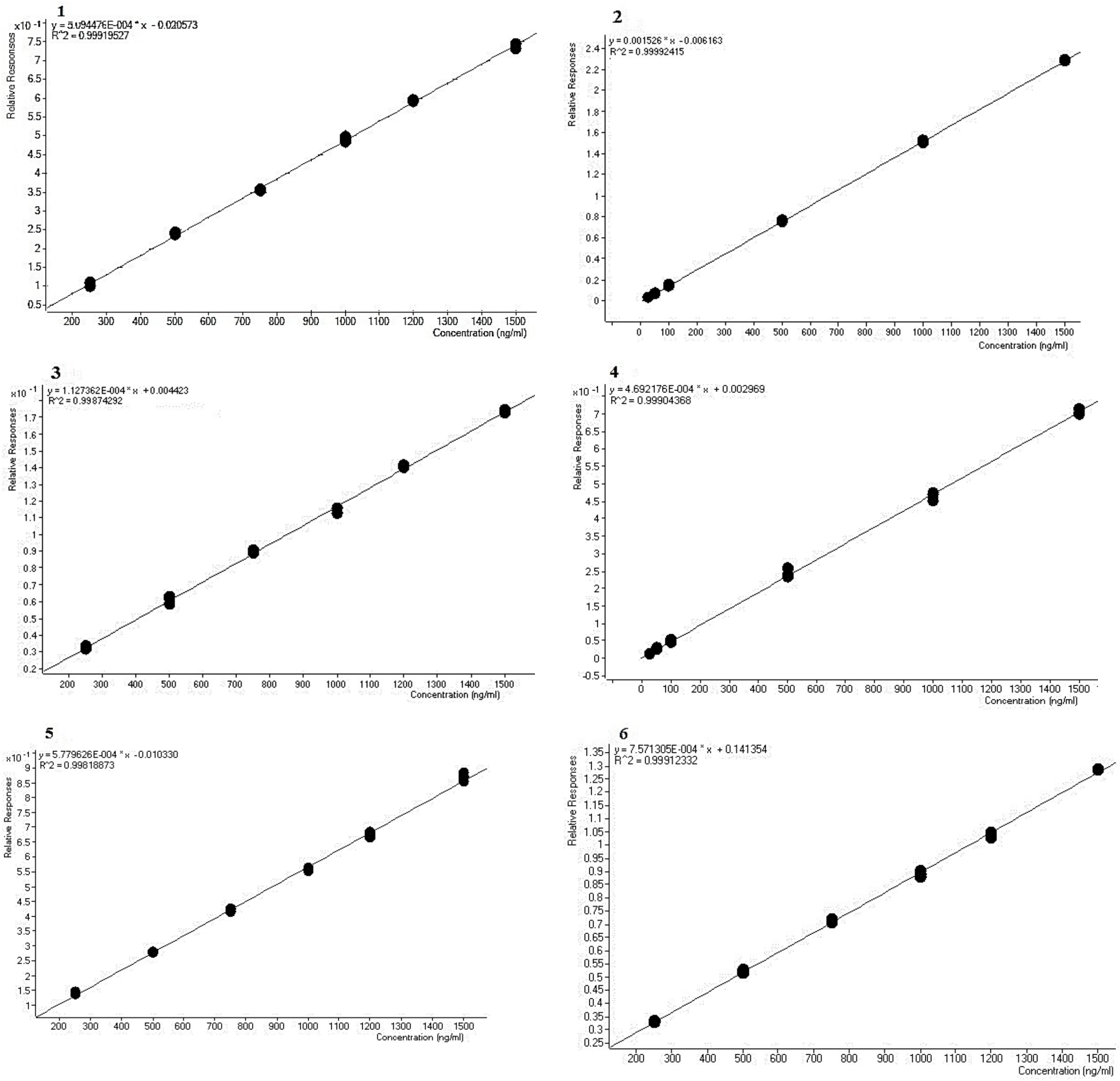

Figure S3. Calibration curve for saponins (**1-6**).

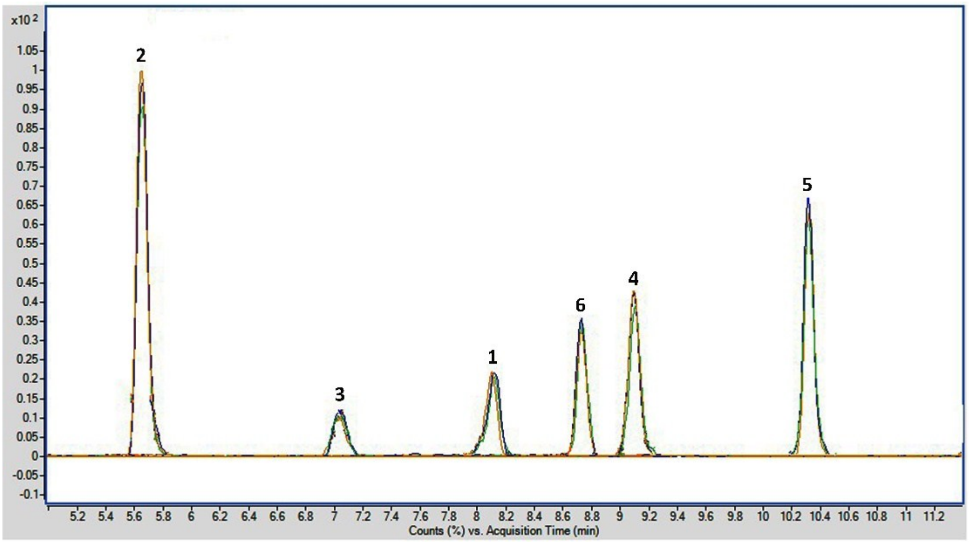

Figure. S4: Overlapping chromatograms depicting reproducibility of six saponins in triplicate analysis.

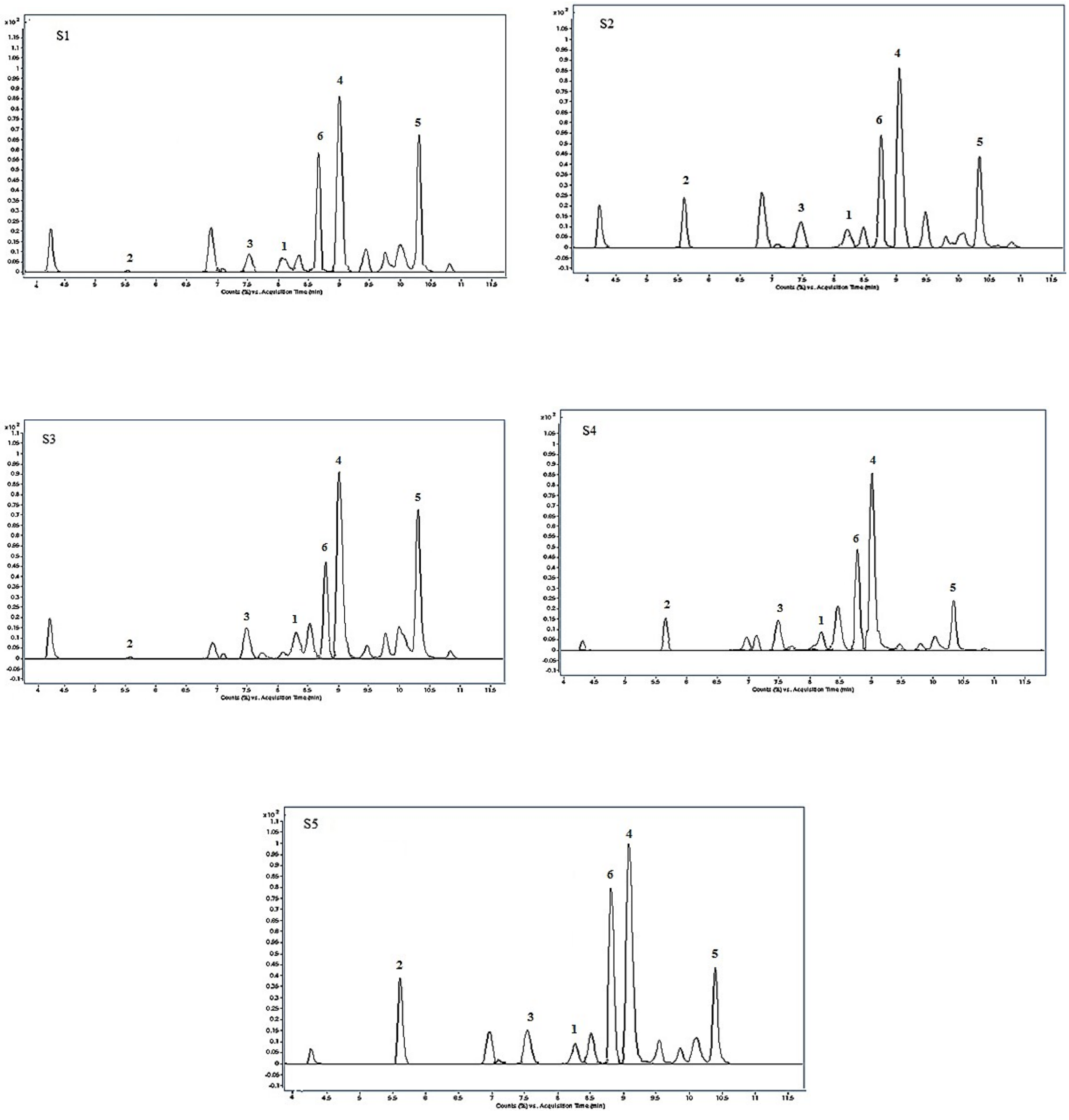

Figure S5. Total ion chromatograms of *F. indica* sample S1-S5.

Table S1. Intra-day and inter-day precisions (RSD %) of the proposed method in three concentration levels.

Name of compounds	Concentration (ng L^{-1})	Intra-day			Inter-day		
		Found (ng mL^{-1})	RSD (%)	Accuracy (%)	Found (ng mL^{-1})	RSD (%)	Accuracy (%)
**1**	450	454±5	1.18	99.07	453±9	1.94	99.11
	600	597±2	0.35	100.49	601±6	1.02	99.88
	900	911±5	0.56	98.72	891±1	0.14	100.98
**2**	150	153±2	1.65	97.93	150±3	2.17	99.77
	450	446.0±0.6	0.15	100.86	451±4	0.93	99.69
	600	596±4	0.62	100.63	599±5	0.86	100.17
	900	901±4	0.44	99.80	902±3	0.30	99.76
**3**	450	444±3	0.59	101.20	448±2	0.34	100.37
	600	598±1	0.24	100.25	612±8	1.29	97.97
	900	891.3±0.1	0.01	100.94	904±3	0.38	99.45
**4**	150	151.0±0.4	0.30	98.89	149±3	1.69	100.40
	450	444±3	0.66	101.28	453±3	0.65	99.16
	600	595±4	0.75	100.78	603±4	0.60	99.43
	900	902±3	0.37	99.71	898±4	0.40	100.11
**5**	450	450±3	0.64	99.89	449±1	0.30	100.26
	600	598±6	1.00	100.19	602±3	0.59	99.62
	900	894±4	0.47	100.63	905±1	0.14	99.41
**6**	450	451±2	0.47	99.61	451±3	0.64	99.62
	600	594±4	0.73	100.92	600±8	1.32	99.93
	900	896±3	0.32	100.39	905±4	0.39	99.41

Table S2. Results of recovery study performed at three spike levels shown in percentage.

Compound	Hyderabad			New sabzi mandi, Karachi			Korangi, Karachi			University of Karachi, Karachi			Super Highway, Karachi		
	**SP1**	**SP2**	**SP3**	**SP1**	**SP2**	**SP3**	**SP1**	**SP2**	**SP3**	**SP1**	**SP2**	**SP3**	**SP1**	**SP2**	**SP3**
**1**	102.8	95.7	94.1	103.2	97.0	97.1	103.9	101.5	97.7	94.3	103.5	96.7	90.0	104.4	107.7
**2**	101.2	90.9	93.2	102.3	95.8	96.9	94.9	96.7	95.4	110.8	109.4	91.0	109.9	103.7	104.1
**3**	103.8	91.7	92.5	97.6	101.4	97.8	105.5	92.0	88.6	114.2	109.7	107.4	108.4	108.9	107.9
**4**	93.5	89.4	86.8	108.9	100.6	93.3	94.7	105.3	94.8	108.8	92.8	104.5	105.9	103.3	106.9
**5**	90.9	94.2	92.5	102.9	102.0	101.6	106.5	116.9	90.1	108.6	90.1	107.0	91.7	102.5	103.2
**6**	89.2	94.6	92.9	95.0	95.5	102.1	105.6	107.2	89.1	89.7	92.3	98.2	107.4	103.2	104.5
